# Transtendinous corticosteroid with contrast injection in trigger finger: a radiological and clinical study

**DOI:** 10.1177/17531934261460230

**Published:** 2026-07-01

**Authors:** Hasan Bitar, Johanna Bergman, Anders Björkman, Joakim Strömberg

**Affiliations:** 1 Institute of Clinical Sciences, Sahlgrenska Academy, University of Gothenburg, Gothenburg, Sweden; 2 Department of Surgery and Orthopaedics, Alingsås Lasarett, Alingsås, Sweden; 3 Department of Radiology, Alingsås Lasarett, Alingsås, Sweden; 4 Department of Hand Surgery, Sahlgrenska University Hospital, Gothenburg, Sweden

**Keywords:** Corticoid injection, radiopaque contrast, tendon sheath, tendovaginitis stenosans, trigger digit, trigger finger, trigger thumb, triggering, trigger phenomenon

## Abstract

**Introduction:**

The aim of this study was to evaluate the distribution of a corticosteroid solution following transtendinous injection at the level of the proximal phalanx. Secondary aims were to explore the patient reported outcomes and analgesic use day-by-day for 6 weeks.

**Methods:**

Twenty trigger digits in 19 patients were injected with a mixture of corticosteroid and radiopaque contrast using a standardized transtendinous technique. Frontal and lateral radiographs obtained immediately after injection were independently assessed by three observers to classify the spread of the solution as intra-sheath, mixed intra-and extra-sheath, or solely extra-sheath.

**Results:**

All 20 digits demonstrated intra-sheath spread of the solution. Thirteen digits showed mixed intra- and extra-sheath distribution, and seven digits demonstrated isolated intra-sheath spread. In eight digits, the solution extended throughout the entire tendon sheath from the palm to the level of the distal phalanx. No cases of isolated extra-sheath injection were observed. Fourteen digits reported total relief of symptoms within 6 weeks. Median number of days to total symptom alleviation was 7 for pain, 8 for triggering and 9 for stiffness. The use of analgesics was low, and 11 patients took none.

**Conclusion:**

Transtendinous injection at the level of the proximal phalanx provides reliable deposition of corticosteroid within the tendon sheath, supporting its use as an acceptable technique for treating trigger finger.

**Level of evidence:**

III prospective cohort study

## Introduction

Corticosteroid injection into the flexor tendon sheath is an effective first-line treatment for trigger finger (Peters-Veluthamaningal et al., 2008). Several injection techniques have been described, most commonly targeting the A1 pulley. However, the accuracy of injections delivered at the A1 pulley level has been questioned. Taras et al. (1998) reported that 17% of injections intended for the tendon sheath at this level resulted in extra-sheath deposition of the injectate, although this did not appear to adversely affect clinical outcomes.

An alternative technique involves injecting at the level of the proximal phalanx using a transtendinous approach, in which the needle is advanced through the flexor tendon to bone and then withdrawn slightly to allow intra-sheath injection (Bitar et al., 2023). This method has been suggested to be less painful for patients (Pataradool and Buranapuntaruk, 2011) and may offer more reliable access to the tendon sheath.

We could not find morphological studies that examined the accuracy of transtendinous injection at the level of the proximal phalanx. Specifically, it is unclear how predictably the injected solution disperses within the tendon sheath and whether extra-sheath deposition occurs.

The aim of this study was to investigate the radiological distribution of a corticosteroid–contrast mixture following transtendinous injection at the level of the proximal phalanx in patients with trigger finger. We sought to determine the frequency of true intra-sheath delivery and to characterize the extent of sheath filling using immediate post-injection radiographs.

## Methods

This was a prospective, morphologic, radiological cohort study conducted between March and December 2024. The STROBE guidelines (Vandenbroucke et al., 2007) were followed in reporting the results of this study. Ethical approval for this study was obtained from the Swedish Ethical Review Authority (Dnr. 2023-04876-01).

### Inclusion criteria

Patients referred to the Orthopaedic Department with trigger digit were assessed and, if eligible for corticosteroid injection, were asked if they would like to participate. Written and oral information was provided, and patients were given time for consideration. In case of approval, written consent was obtained, and baseline data were collected.

The inclusion criteria were adults (>18 years) with triggering in a single digit with stiffness and/or tenderness and pain over the A1 pulley of the affected digit. Exclusion criteria were any previous treatment or operation on the affected digit and limited range of motion due to any other pathological condition.

All patients were treated and assessed by the same researcher (HB).

### Injection technique

One millilitre of a corticosteroid solution already mixed by the manufacturer containing Depo-Medrol^®^ lidocaine 40 mg/ml+10 mg/ml (Pfizer, Stockholm, Sweden) with 0.5 ml radiopaque dye Omnipaque-300 (GE Healthcare AS, Oslo, Norway) was injected in the proximal phalanx using a transtendinous approach (Figure 1). The injection site was located in the centre of the palmar aspect of the proximal phalanx in the midline between the flexion creases. The needle was inserted through skin, subcutaneous tissue, tendon sheath and tendon until bone was reached. Gentle pressure was applied on the syringe as it was retracted slightly to enable free flow of the total volume of injectate into the tendon sheath.

**Figure 1.**
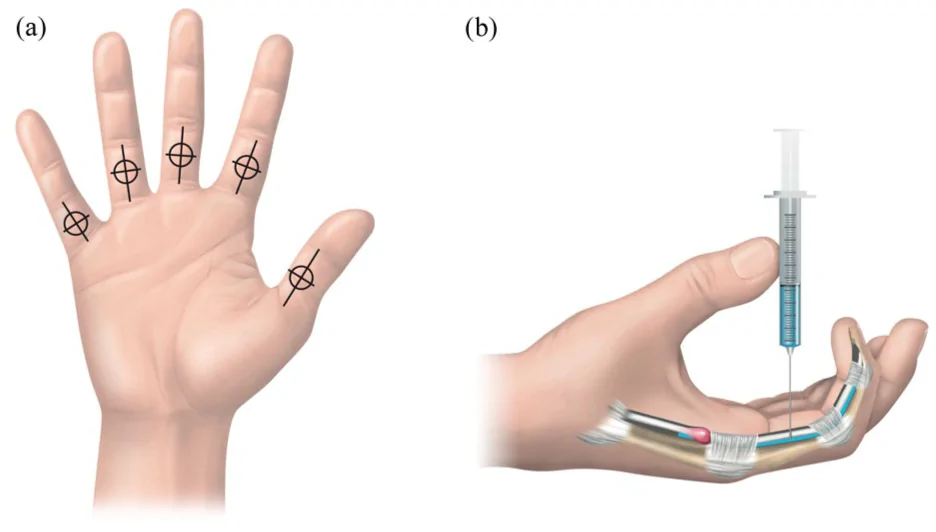
The transtendinous injection technique. (a) The injection site in the centre of the palmar aspect of the proximal phalanx. (b) The needle is inserted until bone is reached, then the syringe is retracted slightly to enable free flow of the corticosteroid and radiologic dye into the tendon sheath.

### Radiologic assessment

After the injection, patients were instructed to actively flex and extend the injected finger five times to promote dispersion of the solution within the tendon sheath. Standard posteroanterior and lateral radiographs of the affected digit were then immediately obtained using conventional digital radiography. The images were stored in the hospital Picture Archiving and Communication System for subsequent evaluation.

Three authors (HB, JB, and JS) conducted a calibration meeting to establish a consensus on the evaluation criteria for the radiographs. Radiographs were classified into one of three categories of radiopaque dye distribution: intra-sheath, mixed intra- and extra-sheath, and extra-sheath. Intra-sheath meant that all radiopaque dye was confined within the tendon sheath proximal and distal to the injection site without subcutaneous infiltration. A small portion of dye confined to the puncture canal was accepted as well as leakage dorsal to the tendon sheath (i.e. between the sheath and the bone). Mixed intra- and extra-sheath was defined as the presence of radiopaque dye both within the tendon sheath proximal and distal to injection site and in the subcutaneous tissue. Extra-sheath meant that no radiopaque dye was visible within the tendon sheath either proximal or distal to the injection site. The three authors (HB, JB, and JS) independently reviewed the radiographs for each patient to determine the inter-rater reproducibility.

### Patient-reported outcomes

Quick Disabilities of the Arm, Shoulder and Hand (DASH) scores (Beaton et al., 2005) were filled in by patients before the injection along with marking a visual analogue scale (VAS) for pain, stiffness, and triggering (range 0–10). The VAS endpoints were defined for each symptom as follows: pain (0, ‘no pain’; 10, ‘worst imaginable pain’); stiffness (0, ‘no stiffness’; 10, ‘maximum stiffness’); and frequency of triggering (0, ‘none’; 10, ‘constantly’).

Patients received follow-up forms at discharge along with a prepaid return envelope. They were instructed to begin recording on the day of injection, documenting the duration of local anaesthesia and VAS scores for pain during injection and after the anaesthesia wore off. Beginning on the first day after injection, patients completed daily self-assessments of VAS scores for pain, stiffness and triggering and recorded daily analgesic use, specifying the type (paracetamol, ibuprofen or other), dosage and frequency of tablets taken. Patients were instructed to return the forms at 6 weeks or once they reported VAS scores of 0 for pain, stiffness and triggering, indicating satisfaction with treatment. Patients were also asked to complete the QuickDASH again before returning the forms.

### Statistical methods

Because this study was exploratory and descriptive in nature, no formal power analysis was performed, and a sample size of 20 digits was deemed appropriate. Normality of continuous variables was assessed using the Shapiro–Wilk test. With the exception of age and number of days for analgesic use, the data were non-normally distributed and are therefore presented as median and range. Inter-rater reliability for the radiographic classification of solution distribution was evaluated using Fleiss’ kappa.

## Results

Twenty patients were invited to participate in the study. One patient declined corticosteroid injection as treatment and preferred to have surgery. Nineteen patients with 20 trigger digits were enrolled in the study between March and December 2024. Seven men and 12 women were included and the mean age was 64 years (SD 13). The most commonly included digits were thumb and ring finger followed by middle finger. Median time of symptoms was 8.5 months. The median QuickDASH score at baseline was 47 (range 7–68).

Conventional radiographs showed an intra-sheath spreading of the injected solution in all patients as well as subcutaneous presence that varied in size in 13 patients. The most common radiological pattern was mixed intra- and extra-sheath spreading (Figure 2), which was found in 13 digits. Seven digits had solely intra-sheath spreading of the solution (Figure 3). Isolated extra-sheath spreading could not be detected in any patient. In eight digits, the solution filled the whole sheath along with the tendon from the palmar fascia to the distal phalanx (Figure 4). No radiographs demonstrated leakage proximal or distal to the sheath, suggesting that the tendon sheath functions as a closed space under these conditions.

**Figure 2.**
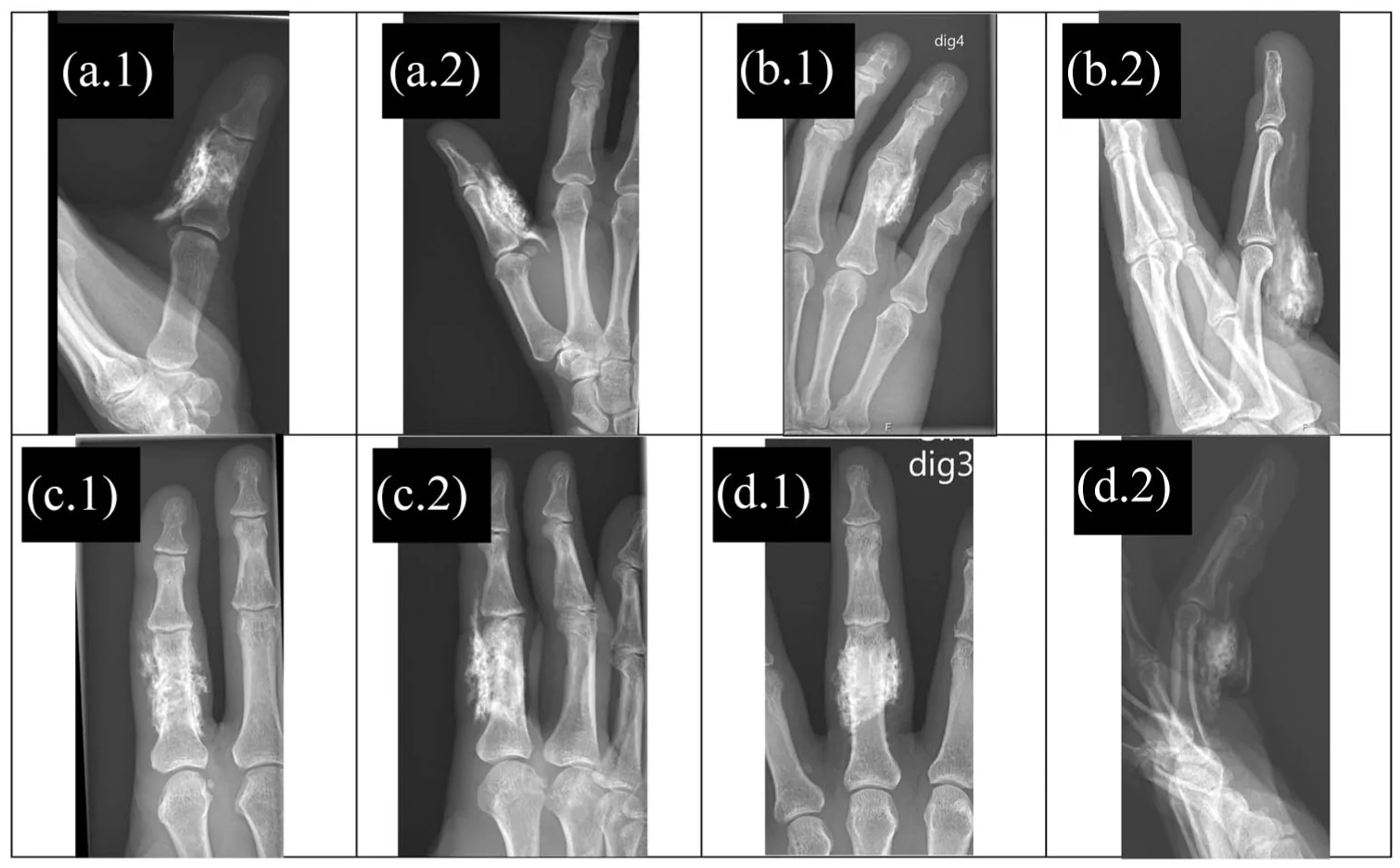
Posteroanterior and lateral finger radiographs showing mixed intra- and extra-sheath spreading of the solution in four patients: (a.1) and (a.2) of patient 3; (b.1) and (b.2) of patient 6; (c.1) and (c.2) of patient 17; and (d.1) and (d.2) of patient 19.

**Figure 3.**
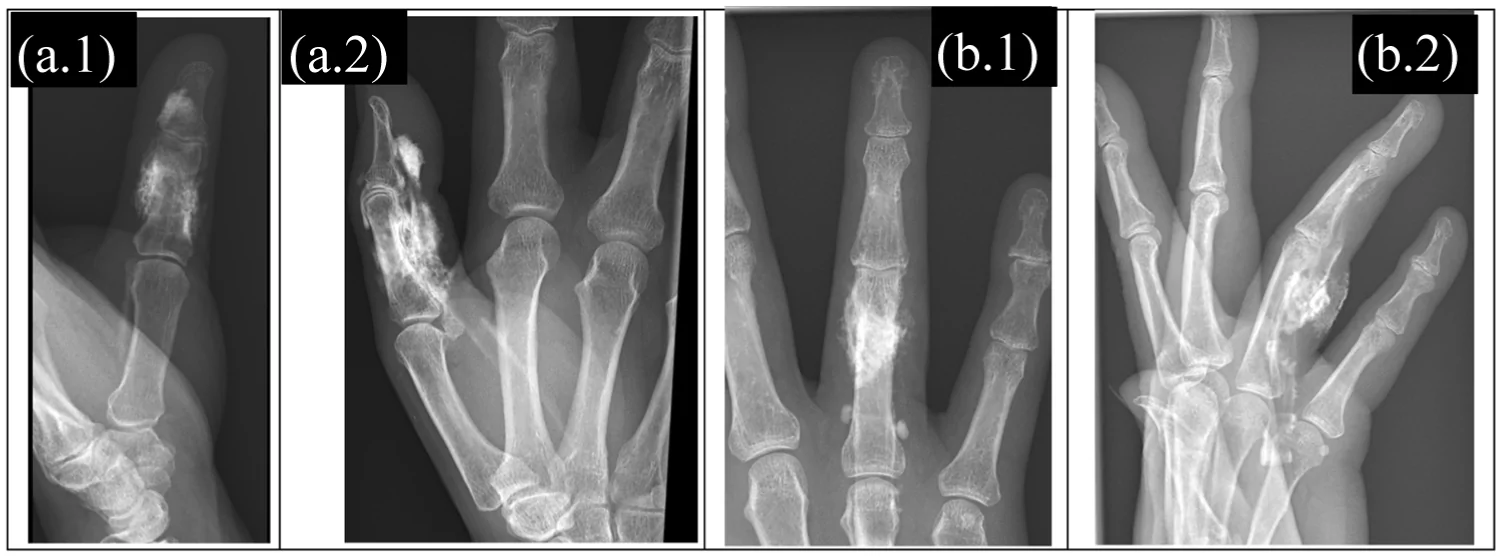
Posteroanterior and lateral finger radiographs of two patients with intra-sheath spreading of the solution: (a.1) and (a.2) of patient 4; and (b.1) and (b.2) of patient 11.

**Figure 4.**
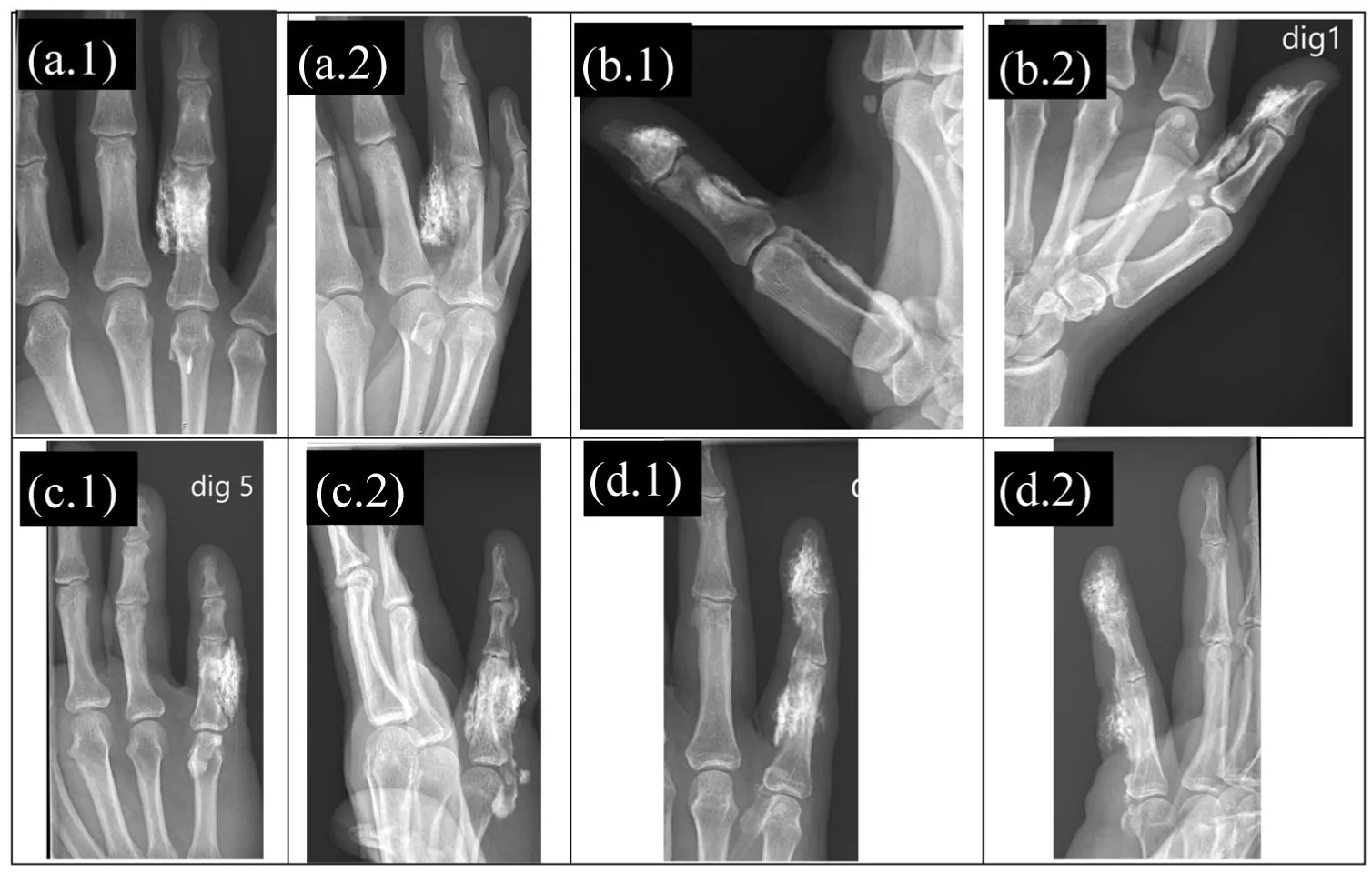
Posteroanterior and lateral radiographs showing the solution filling the whole tendon sheath in four patients: (a.1) and (a.2) of patient 1; (b.1) and (b.2) of patient 2; (c.1) and (c.2) of patient 12; and (d.1) and (d.2) of patient 16.

Agreement among raters was achieved in 16 of the 20 cases. For the four cases without complete consensus, the category endorsed by at least two of the three raters was used. Inter-rater reliability, calculated using Fleiss’ kappa, was 0.72 (95% CI 0.47 to 0.97; *p* < 0.001), indicating substantial agreement among the three authors according to the criteria of Landis and Koch (1977).

Out of 20 digits, 14 reported total relief of all symptoms within 6 weeks. The median numbers of days to total symptom relief were: 7 days for pain (range 1–36, *n* = 16), 8 days for triggering (range 1–16, *n* = 15) and 9 days for stiffness (range 1–40, *n* = 15). Median QuickDASH at satisfaction was 10 (range 0–55).

Eleven patients (12 digits) did not use any analgesics, one used paracetamol 500 mg/codeine 30 mg for one day (eight tablets) and seven patients used over-the-counter paracetamol 500 mg and/or ibuprofen 400 mg (one or two tablets a day) for a mean of 4 days (SD 3).

## Discussion

This study demonstrated that transtendinous corticosteroid injection at the level of the proximal phalanx consistently delivered the injected solution into the flexor tendon sheath. All digits showed intra-sheath distribution on radiographs, and no cases of isolated extra-sheath deposition were identified. These findings suggest that this technique provided reliable access to the tendon sheath.

In a randomized control trial, Taras et al. (1998) compared the clinical results of injection at the A1 pulley for trigger finger in 48 patients (52 fingers) who had an intra-sheath injection and 48 patients (55 fingers) who had a subcutaneous injection. A radiopaque dye was added to the solution and radiographs were taken. In the attempted intra-sheath group, the steroid solution was completely within the tendon sheath in 19 (37%) digits, both in the sheath and in the subcutaneous tissues in 24 (46%) digits, and the solution failed to enter the tendon sheath in nine digits (17%). Clinical outcomes were the same regardless of the location of the injectate. Cadaveric work in 60 fingers by Binz et al. (2023) demonstrated that transtendinous injection at the level of the proximal phalanx resulted in intra-sheath or mixed distribution in 95% of cases, with only one digit showing purely extra-sheath spread. The 100% intra-sheath involvement observed in our cohort supports the reliability of this technique *in vivo*.

Pataradool and Buranapuntaruk (2011) reported in a randomized controlled trial with 40 trigger fingers a mean VAS score for pain of 3 directly after injection at the level of the proximal phalanx and a mean VAS of 7 directly after A1 pulley injection (*p* < 0.001). The present study provided morphological confirmation that the technique reliably deposits medication within the target compartment. Our previous randomized trial in 106 patients (Bitar et al., 2023) could not detect any significant difference in the clinical outcome (VAS for pain, stiffness and triggering and QuickDASH score) between injection at the A1 pulley or over the proximal phalanx. Hence, injection at the level of the proximal phalanx may be less painful but equally effective.

Radiographs frequently showed a mixed pattern with both intra- and extra-sheath spread. This finding does not necessarily indicate technical error, as a small subcutaneous component may result from needle passage or withdrawal and does not preclude full intra-sheath filling.

The study has limitations. The addition of radiopaque dye could theoretically alter the properties of the injection; however, previous work has shown that corticosteroid–contrast mixtures are chemically stable (Shah et al., 2009). The sample size was modest, although we believe that it was adequate for the descriptive aim of the study. Furthermore, radiographs were assessed by one of the authors who also injected the patients, and this is a potential source of bias. Calibration among the three assessors and the establishment of predefined criteria before individual assessment was conducted to reduce this risk. Finally, there was 20% disagreement between raters on radiographic assessment. This may be explained by the difficulty in assessing the subcutaneous infiltration from plain radiographs. There was, however, no digit assessed as solely extra-sheath distribution. Use of ultrasound might be more effective in distinguishing subcutaneous infiltration.
